# Efficacy and safety of acupuncture combined with Xuefu Zhuyu Decoction on major adverse cardiovascular events after percutaneous coronary intervention: A protocol for systematic review and meta-analysis

**DOI:** 10.1097/MD.0000000000031735

**Published:** 2022-11-18

**Authors:** Qingyuan Ma, Zhimei Cai, Lan Sui, Xiaoxia Wang

**Affiliations:** a Department of Geriatrics, The First People’s Hospital of Lianyungang, The Affiliated Lianyungang Hospital of Xuzhou Medical University, The First Affiliated Hospital of Kangda College of Nanjing Medical University, Lianyungang Clinical College of Nanjing Medical University, Lianyungang, China; b Department of Hematology, The First People’s Hospital of Lianyungang, The Affiliated Lianyungang Hospital of Xuzhou Medical University, The First Affiliated Hospital of Kangda College of Nanjing Medical University, Lianyungang Clinical College of Nanjing Medical University, Lianyungang, China; c The Second Acupuncture Department, Shaanxi Provincial Hospital of Chinese Medicine, Xi’an, China; d The First Acupuncture Department, Shaanxi Provincial Hospital of Chinese Medicine, Xi’an, China.

**Keywords:** acupuncture, major adverse cardiovascular events, meta-analysis, percutaneous coronary intervention, protocol, Xuefu Zhuyu Decoction

## Abstract

Percutaneous coronary intervention (PCI) is an effective treatment for coronary heart disease (CHD). With the merits of small trauma and high success rate, PCI can promote the fast recovery of CHD patients and greatly improve their prognosis and quality of life. However, because PCI does not eliminate the pathogenic factors that lead to atherosclerosis, major adverse cardiovascular events (MACEs) often occur after PCI. These events have become one of the principal factors affecting the long-term outcome of patients after PCI. In China, increasing attention is paid to the use of acupuncture combined with Xuefu Zhuyu Decoction (XFZYD) for the treatment of post-PCI MACEs in clinical practice. Nevertheless, this treatment approach still lacks evidence-based medical evaluation. In this study, a meta-analysis was conducted to evaluate the effectiveness and safety of acupuncture combined with XFZYD in the treatment of MACEs after PCI.

**Methods:**

Randomized controlled trials on the efficacy and safety of acupuncture combined with XFZYD for the treatment of MACEs after PCI were retrieved from CNKI, WanFang, PubMed, Embase, Cochrane Library, Google Scholar and Web of Science databases from the time of database establishment to October 2022. The papers were screened strictly according to the inclusion and exclusion criteria, and the quality of the included studies was assessed using the Risk of Bias 2 (RoB 2) tool. Raw data were extracted from the studies and then a meta-analysis was made using RevMan 5.3 software.

**Results:**

The results of this meta-analysis will be submitted to a peer-reviewed journal for publication.

**Conclusion:**

This study will summarize the latest evidence for the efficacy and safety of acupuncture combined with XFZYD in the treatment of MACEs after PCI.

REGISTRATION NUMBER: CRD42022365657.

## 1. Introduction

Coronary heart disease (CHD) is characterized by myocardial ischemia, hypoxia and necrosis, which are resulted from atherosclerosis and spasm induced luminal narrowing or even blockage, with chest pain as the main clinical symptom.^[1,2]^ The morbidity and mortality rates of CHD are still ascending in China, and CHD is the leading cause of death from disease among the population.^[3]^ Currently, the treatment methods for CHD mainly include drug therapy, percutaneous coronary intervention (PCI), coronary artery bypass grafting, etc.^[4–6]^ PCI has become an important clinical modality for the treatment of CHD because of its advantages of simple procedure, rapid onset of action and minimal trauma.^[7,8]^ During the promotion of PCI in clinical practice, cardiovascular events such as angina, acute thrombosis, and reperfusion injury have been observed in patients after PCI.^[9]^ Major adverse cardiovascular events (MACEs) after PCI have a great impact on the long-term outcome of patients.^[10,11]^ Intensive treatment with conventional western drugs after PCI has some shortcomings, such as side effects of drugs and expensive medical costs.^[12,13]^

Chinese medicine has been shown capable of alleviating clinical symptoms, increasing exercise tolerance, reducing angina attacks, preventing restenosis after intervention, and even improving long-term prognosis in the treatment of CHD.^[14,15]^ In the traditional Chinese medicine science, CHD is classified as “chest paralysis and heart pain,” which is a syndrome of deficiency in origin and excess in superficiality.^[16]^ In clinical practice, CHD patients are often treated by tonifying qi, invigorating the circulation of blood and removing blood stasis after PCI.^[17]^

Xuefu Zhuyu Decoction (XFZYD) has the effects of promoting blood circulation, eliminating blood stasis, activating vital energy to stop pain, and reinforcing functional activities of the heart, so it can greatly relieve angina pectoris after PCI.^[18,19]^ XFZYD is a traditional classic soup for the treatment of chest paralysis, originated from the book “Yilin Gaicuo.”^[20]^ Modern pharmacological analysis suggests that XFZYD can improve myocardial microcirculation perfusion and myocardial contractility, effectively inhibit platelet aggregation, dilate coronary arteries and peripheral vessels, prevent the formation of atherosclerosis, and ameliorate myocardial ischemia and hypoxia.^[21]^

Acupuncture combined with XFZYD for treating CHD patients after PCI is superior to conventional western drug therapy in the clinical efficacy. However, evidence from a single randomized controlled trial is relatively insufficient, and no relevant systematic evaluation has been published. Therefore, in this study, a meta-analysis was made to evaluate the efficacy and safety of acupuncture combined with XFZYD for the treatment of MACEs after PCI. The results of this paper provide a basis for clinical application of the proposed treatment method.

## 2. Method

This systematic review protocol has been registered in the PROSPERO (CRD42022365657). We will follow recommendations outlined in the preferred reporting items for systematic reviews and meta-analysis protocol (PRISMA-P) statement guidelines.^[22]^

### 2.1. Study selection according to PICOs criteria

#### 2.1.1. Participants.

All patients with CHD after PCI were included. CHD diagnostic criteria were published guidelines, principles, standards and other accepted criteria.

#### 2.1.2. Interventions and comparison.

The experimental group received acupuncture combined with XFZYD, while the control group was treated with conventional western medicine. The conventional western medicine used included aspirin, clopidogrel, tigretol and other drugs for anti-platelet aggregation, heparin anticoagulation, statin lipid lowering, anti-inflammation.

#### 2.1.3. Outcome measures.

MACEs were recurrent angina after PCI, restenosis after PCI, myocardial infarction, severe arrhythmias, heart failure, and cardiac death.

Secondary outcome indicators included overall efficiency, angina efficiency, ECG efficiency, left ventricular ejection fraction, and adverse events.

#### 2.1.4. Study design.

Randomized controlled trials that covered the efficacy and safety of acupuncture combined with XFZYD for treating MACEs after PCI were selected.

### 2.2. Exclusion criteria

Studies with an extremely small sample size (<30 cases); studies with an incomparable baseline or studies that did not clearly state the baseline was comparable; studies that adopted other traditional Chinese medicine methods such as acupressure in the test group; studies that employed Chinese medicine or traditional Chinese medicine therapy in the control group; and studies with unavailable full text and unusable data. Only one of different papers that used the same data was included.

### 2.3. Search strategy

Randomized controlled trials were extracted from CNKI, WanFang, PubMed, Embase, Cochrane Library, Google Scholar, and Web of Science from the time of library establishment to October 2022. All studies on the efficacy and safety of acupuncture combined with XFZYD for MACE treatment after PCI were collected. PubMed was taken as an example, and its search strategy is shown in Table 1.

**Table 1:** PubMed search strategy.

Number	Search terms
#1	Coronary Disease[MeSH]
#2	Coronary Heart Disease[Title/Abstract]
#3	Coronary Diseases[Title/Abstract]
#4	Coronary Heart Diseases[Title/Abstract]
#5	Disease, Coronary[Title/Abstract]
#6	Disease, Coronary Heart[Title/Abstract]
#7	Diseases, Coronary[Title/Abstract]
#8	Diseases, Coronary Heart[Title/Abstract]
#9	Heart Disease, Coronary[Title/Abstract]
#10	Heart Diseases, Coronary[Title/Abstract]
#11	or/1-10
#12	Percutaneous Coronary Intervention[MeSH]
#13	Percutaneous Coronary Revascularization[Title/Abstract]
#14	Coronary Intervention, Percutaneous[Title/Abstract]
#15	Coronary Interventions, Percutaneous[Title/Abstract]
#16	Coronary Revascularization, Percutaneous[Title/Abstract]
#17	Coronary Revascularizations, Percutaneous[Title/Abstract]
#18	Intervention, Percutaneous Coronary[Title/Abstract]
#19	Interventions, Percutaneous Coronary[Title/Abstract]
#20	Percutaneous Coronary Interventions[Title/Abstract]
#21	Percutaneous Coronary Revascularizations[Title/Abstract]
#22	Revascularization, Percutaneous Coronary[Title/Abstract]
#23	Revascularizations, Percutaneous Coronary[Title/Abstract]
#24	or/12-23
#25	Xuefu Zhuyu[Title/Abstract]
#26	Circulation Plus Decoction[Title/Abstract]
#27	or/25-26
#28	Randomized Controlled Trials as Topic[MeSH]
#29	Clinical Trials, Randomized[Title/Abstract]
#30	Controlled Clinical Trials, Randomized[Title/Abstract]
#31	Trials, Randomized Clinical[Title/Abstract]
#32	Random*[Title/Abstract]
#33	or/28-32
#34	#11 and #24 and #27 and #33

### 2.4. Data collection and analysis

#### 2.4.1. Selection of studies.

**Figure 1.**
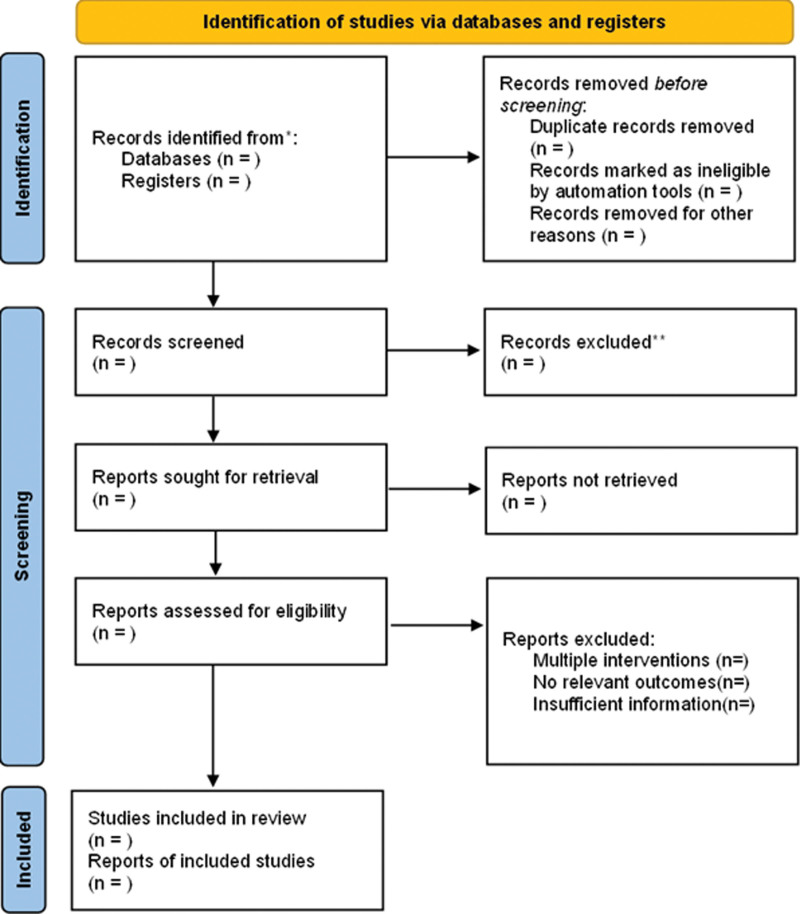
Flow diagram of literature retrieval.

Two investigators read the title and abstract of collected studies for initial screening separately according to the established inclusion and exclusion criteria. After further examining the full text of the literature that met the inclusion criteria, the investigators decided which papers to include. Disagreements between the two investigators about the included literature were discussed, or a third party was consulted to assist in the judgment. The selection process was summarized using the PRISMA flowchart. Details of the study selection process are shown in Figure 1.

#### 2.4.2. Data extraction and management.

Two investigators were responsible for data extraction and management. Data extracted included the basic information of the included literature, study object, sample size, study period, diagnostic method, study design method, cardiac function and MACE. After data extraction, 2 investigators cross-checked the extraction results. In case of disagreement, they discussed and consulted with a third investigator.

#### 2.4.3. Dealing with missing data.

Corresponding authors would be contacted if complete texts or relevant data were not available. If missing data could not be obtained, the study was excluded from the analysis.

#### 2.4.4. Assessment of risk of bias.

The Risk of Bias 2 (RoB 2) tool was used to evaluate the quality of papers included. This tool covers five domains: the bias caused by the randomization process, deviations from the established intervention, missing outcome data, outcome measures, and selective reporting of outcomes. Each domain was evaluated to be at high risk, medium risk, or low risk. The five domains were assessed comprehensively to produce an overall evaluation result.

#### 2.4.5. Measures of treatment effect.

Standardized mean difference (SMD) and relative risk (RR) were taken as an effect indicator of continuous and dichotomous variables, respectively. Both indicators were expressed with 95% confidence intervals (CI).

#### 2.4.6. Assessment of heterogeneity.

I2 was used to determine the heterogeneity between studies. *P* > .1 and *I*^2^ < 50% indicate that the heterogeneity can be ignored. *P* < .1 and *I*^2^ > 50% represent a significant heterogeneity among the included studies.

#### 2.4.7. Assessment of reporting bias.

Funnel plot was used to verify whether there was publication bias.^[23]^

#### 2.4.8. Data synthesis.

Review Manager (RevMan 5.3, Cochrane Collaboration, Nordic Cochrane Center, Copenhagen, Denmark) software was employed for statistical analysis. A fixed effects model would be adopted if no statistical heterogeneity was observed between the results (*P* > .1 and *I*^2^ < 50%). Otherwise, a random-effects model would be used (*P* < .1 and *I*^2^ > 50%).

#### 2.4.9. Subgroup analysis.

Subgroup analysis would be performed according to the treatment course and the type of acupuncture.

#### 2.4.10. Sensitivity analysis.

Sensitivity analysis was conducted to check the robustness of study findings by excluding studies with low methodological quality and large sample size.

#### 2.4.11. Evaluation of evidence quality.

To evaluate the quality of evidence for each outcome indicator, the risk of bias, inconsistency, indirectness, imprecision, and publication bias in the study design were investigated using the Grading of Recommendations Assessment, Development and Evaluation (GRADE) method.

#### 2.4.12. Ethics and dissemination.

Since this study does not involve patient privacy, ethical approval is not required. The findings of this study will be published in a peer-reviewed journal.

## 3. Discussion

Current main treatments for patients after PCI are anticoagulation, antiplatelet aggregation and lipid-lowering medication.^[24]^ However, conventional western drugs have a single target, so they cannot effectively improve the clinical symptoms of patients and require long-term administration. Besides, western medicine may produce adverse effects, and can hardly reduce the occurrence of long-term MACEs.^[13,25]^ Over the past years, Chinese medicine research has made great progress in various aspects. Several studies have established that the combination of conventional western medicine with Chinese herbal medicine can not only further improve clinical symptoms but also lower the incidence of long-term MACEs.^[13,26]^ In this study, the efficacy and safety of acupuncture combined with XFZYD for the treatment of MACEs after PCI are systematically evaluated so as to provide evidence-based medical guidance for this application in clinical practice.

## Author contributions

**Conceptualization:** Qingyuan Ma, Xiaoxia Wang.

**Data curation:** Zhimei Cai.

**Formal analysis:** Lan Sui.

**Funding acquisition:** Xiaoxia Wang.

**Software:** Qingyuan Ma.

**Supervision:** Xiaoxia Wang.

**Writing – original draft:** Qingyuan Ma, Xiaoxia Wang.

**Writing – review & editing:** Qingyuan Ma, Xiaoxia Wang.
